# Quantitative mass spectrometry analysis of the injured proximal and distal human digital nerve ends

**DOI:** 10.3389/fnmol.2024.1425780

**Published:** 2024-07-02

**Authors:** Drifa Frostadottir, Charlotte Welinder, Raquel Perez, Lars B. Dahlin

**Affiliations:** ^1^Department of Translational Medicine – Hand Surgery, Lund University, Malmö, Sweden; ^2^Department of Hand Surgery, Skåne University Hospital, Malmö, Sweden; ^3^Faculty of Medicine, Department of Clinical Sciences, Mass Spectrometry, Lund University, Lund, Sweden; ^4^Unit for Social Epidemiology, Department of Clinical Sciences, Malmö, Lund University, Malmö, Sweden; ^5^Department of Biomedical and Clinical Sciences, Linköping University, Linköping, Sweden

**Keywords:** digital nerve injury, quantitative mass spectrometry, proteomics, peripheral nerve injury, nerve injury pathways, signal transduction, extracellular matrix

## Abstract

**Introduction:**

Proteomic analysis of injured human peripheral nerves, particularly focusing on events occurring in the proximal and distal nerve ends, remains relatively underexplored. This study aimed to investigate the molecular patterns underlying a digital nerve injury, focusing on differences in protein expression between the proximal and distal nerve ends.

**Methods:**

A total of 26 human injured digital nerve samples (24 men; 2 women; median age 47 [30–66] years), harvested during primary nerve repair within 48 h post-injury from proximal and distal nerve ends, were analyzed using mass spectrometry.

**Results:**

A total of 3,914 proteins were identified, with 127 proteins showing significant differences in abundance between the proximal and the distal nerve ends. The downregulation of proteins in the distal nerve end was associated with synaptic transmission, autophagy, neurotransmitter regulation, cell adhesion and migration. Conversely, proteins upregulated in the distal nerve end were implicated in cellular stress response, neuromuscular junction stability and muscle contraction, neuronal excitability and neurotransmitter release, synaptic vesicle recycling and axon guidance and angiogenesis.

**Discussion:**

Investigation of proteins, with functional annotations analysis, in proximal and the distal ends of human injured digital nerves, revealed dynamic cellular responses aimed at promoting tissue degeneration and restoration, while suppressing non-essential processes.

## 1 Introduction

Peripheral nerve injuries can lead to significant functional impairments, severely affecting patients’ quality of life. Understanding the immediate molecular mechanisms that govern nerve injury response and regeneration is crucial for developing effective therapeutic surgical and pharmacological interventions. Data on up- and down-regulated proteins from experimental studies must also be confirmed in human studies (Krishnan et al., 2024). Following a nerve transection, the proximal nerve end around the site of the lesion and the entire distal nerve end undergoes distinct and extensive biological processes. From the proximal nerve end, the axons attempt to regenerate across the site of injury and into the distal nerve end, where Wallerian degeneration is rapidly initiated to prepare the environment for potential regeneration. Despite the clinical importance of these processes, comprehensive proteomic differences between these nerve ends have not been extensively studied in a well-defined time perspective, nor have they been adequately related to findings in experimental studies.

The peripheral nervous system possesses the ability to undergo self-regeneration following an injury, where the efficiency may depend on the extent and location of the injury, the surgical methods of repair, or reconstruction as well as the timing of the surgical procedure. However, the functional recovery is often slow and incomplete due to its reliance on the synthesis and transport of intracellular substances in the neuron that are crucial for axonal outgrowth, which must occur along the entire distal nerve end to the target. The regeneration process also involves expression of substances by for example Schwann cells (SC) and recruited macrophages (Court et al., 2008; Jessen and Mirsky, 2016; Clements et al., 2017). The regenerative capacity of axons decreases with increasing distance from the injury site, suggesting the presence and impact of crucial signals originating particularly from the Schwann cells (SC) in the distal nerve end. Timing of surgery, related to utilization of up- and downregulated molecules by the injury over time, plays an important role in guiding axonal outgrowth (Danielsen et al., 1983; Huebner and Strittmatter, 2009).

Although individual proteins and pathways involved in nerve regeneration have been investigated in mice and rats (Dahlin, 2023; Krishnan et al., 2024), there is a lack of comprehensive proteomic analysis comparing the protein profiles of proximal and distal nerve ends post-injury in humans, where the proximal nerve end is considered to exhibit molecular alterations. This gap of knowledge limits our understanding of the molecular environment and sophisticated regulatory mechanisms, particularly in the distal nerve end, which are essential for devising targeted therapeutic strategies and for interpreting findings from experimental studies.

From the proximal injured nerve end, neurons immediately after injury, attempt to regrow their axons across the site of injury and down into the distal nerve end to their target tissue, guided by dedifferentiated SC that become activated and proliferate distal to the injury, forming structures known as bands of Büngner (Nocera and Jacob, 2020). Schwann cells undergo a transition from a myelinating state to a growth-supportive state after loss of axonal contact. Changes occurs also in the SC related to the non-myelinated nerve fibers (Saito et al., 2010). In both the neuronal cell body and SC, several signaling pathways are initiated to facilitate the regenerative response (Bolívar et al., 2020), which is supported and facilitated by other key players, such as the crucial macrophages (Zigmond and Echevarria, 2019). The changes in the proximal nerve end usually involve only a limited distance from the site of injury, extending to the nearest node of Ranvier in myelinated nerve fibers, resulting in limited alteration of the proteomic pattern. Thus, understanding the short-term proteomic differences between the proximal and distal nerve in humas is a suitable model to identify proteins that are differentially expressed, potentially revealing novel targets for therapeutic intervention. Proteins uniquely up- or downregulated in either nerve end can serve as biomarkers to monitor the progression of nerve degeneration and regeneration and relate to the effectiveness of treatments. Analyzing the early events, such as within the first days after injury, in the proximal and distal nerve ends is an initial step using proteomics in humans with a well-defined injury.

The distal nerve end is believed to release trophic factors and other signaling molecules that promote axonal regeneration and guide growing axons towards their targets (Lundborg, 1987; Neurotropism and nerve growth, 1987). Furthermore, the distal nerve end may undergo changes over time following injury, such as alterations in gene expression of the key players or composition of extracellular matrix, including the inflammatory response and also the presence of pro-healing macrophages (Zigmond and Echevarria, 2019) with risk of formation of scarring, which affect its ability to support axonal outgrowth over longer distances. As the distance between the proximal and distal nerve ends increases, the concentration of signaling molecules may decrease or become insufficient to sustain robust axonal outgrowth, despite formation of a formed and crucial fibrin matrix with macrophages between the nerve ends. Such a fibrin matrix is influenced by the distance between the nerve ends (Zhao et al., 1993). Additionally, the time frame during which these signals remain active may be limited, further limiting the window of opportunity for successful regeneration (Sulaiman and Gordon, 2013).

In recent years, significant progress has been made in understanding the specific signaling molecules emitted by the injured peripheral nerves with mass spectrometry techniques. Neurotrophic factors, extracellular matrix molecules, and signaling pathways have emerged as key players in promoting axonal outgrowth and survival (Rozenbaum et al., 2018; Vergara et al., 2018; He et al., 2021) Knowledge has been raised from animal experimental studies, using both mice and rats along with *in vitro* studies (Raivich, 2011; Jessen and Mirsky, 2019; Jessen and Mirsky, 2021). However, studies with focus on proteomic changes at the tip of the injured human proximal and distal nerve ends are limited.

Thus, understanding the differences between the proximal and distal nerve ends after injury is vital for crafting effective strategies to enhance nerve regeneration. While alterations are thought to be limited in the proximal nerve end after nerve injury, the distal end may undergo significant cellular and molecular alterations with the intention to clear the path for the outgrowing axons. The aim of the study is to investigate the molecular changes underlying a human digital nerve injury, focusing on changes in protein expression in the distal nerve end compared to the proximal nerve end of human digital nerves.

## 2 Materials and methods

### 2.1 Ethical approval

The study has been approved by the Regional Ethical Review Board in Lund, Sweden (no 311/2016). All study participants provided informed written consent. All procedures were carried out in line with relevant current guidelines and regulations. Prior to surgery an informed consent was retrieved from the donor. The research was conducted in accordance with the principles of the Helsinki Declaration.

### 2.2 Subjects

Patients with a transected digital nerve injury, where the proximal and the distal digital nerve ends could be visualized, and a primary surgical intervention was performed within 48 h from injury were included in the study. Only patients with a clearcut digital nerve injury that did not require extensive resection were included, while patients undergoing a nerve reconstruction procedure were not included. A short segment of the injured proximal and distal end of the digital nerve was removed, during an acute surgical procedure at the Department of Hand Surgery in Malmö, Sweden, as part of preparing the injured nerve for a direct nerve repair with sutures. Thereby, only nerve injuries with a limited impact on the nerve ends were included. The resected nerve pieces were collected and stored at −80°C until further analysis.

**Figure 1:**
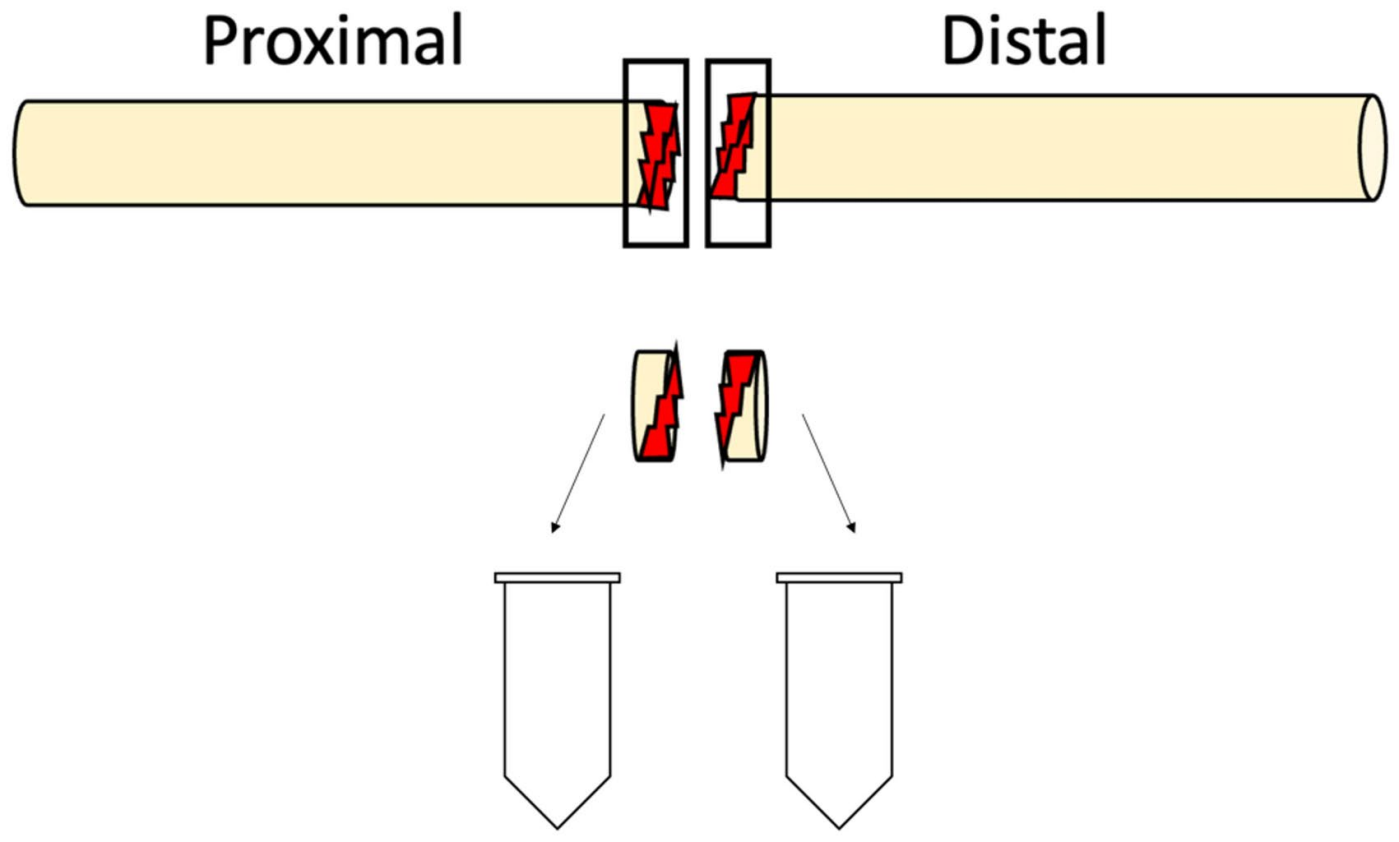
A schematic drawing of the harvesting procedure of nerve tissue from the transected digital nerve performed within 48 h after injury. The injured segment of the proximal and distal end of the digital nerve were collected and stored at −80°C.

The study included 19 individuals (men *n* = 24; women *n* = 2; median age 47 [interquartile range; IQR 30–66]), four individuals had multiple digital nerve injuries leading to a final inclusion of 26 paired digital nerve injuries, each containing a proximal and a distal nerve end harvested during the surgical repair, subsequently leading to the analysis of 52 nerve ends (Figure 1).

### 2.3 Sample preparation

Samples were prepared as previously described (Frostadottir et al., 2024). Briefly, fresh frozen human digital nerve tissue pieces were homogenized using a Bullet Blender Storm Pro (BT24M, Next Advance, Inc. Troy, NY, United States) first with Ripa buffer, and then followed by 8 M Urea. Enzyme digestion was performed by LysC for 3 h at 37 C followed by trypsination overnight at 37 C.

#### 2.3.1 LC–MS analysis

The samples were analyzed on an Orbitrap Eclipse Tribrid mass spectrometer (Thermo Fischer Scientific) coupled with an Ultimate 3,000 RSLCnano system (Thermo Fischer Scientific). Two-column setup was used on the HPLC system and peptides were loaded into an Acclaim PepMap 100 C18 precolumn (75 μm x 2 cm, Thermo Scientific, Waltham, MA) and then separated on an EASY spray column (75 μm x 25 cm, nanoViper, C18, 2 μm, 100 Å) with the flow rate 300 nL/min. The column temperature was set at 45°C. Solvent A (0.1% FA in water) and solvent B (0.1% FA in 80% ACN) were used to create a non-linear gradient to elute the peptides. For the gradient, the percentage of solvent B was maintained at 2% for 4 min, increased from 2 to 25% for 100 min and then increased to 40% for 20 min and then increased to 95% for 1 min and then kept at 95% for another 5 min to wash the column.

The Eclipse was operated in the data-independent acquisition mode. Full MS survey scans from m/z 350–1,450 with a resolution 120,000 were performed in the Orbitrap detector. The automatic gain control (AGC) target was set to 1 × 10^**6**^ with the maximum injection time of 50 ms. One segment for MS1 was kept constant. Forty-four windows with isolation width of 25 m/z. were acquired for MS2 with the resolution of 30,000. The normalized collision energy for HCD and the AGC target for MS2 were 30 and 1 × 10^**6**^, respectively. The maximum injection time was set to 54 ms.

#### 2.3.2 Data analysis

A total of 52 DIA runs were loaded in Spectronaut™ (version 18.4, Biognosys AG) and the protein search was performed using the human protein data base downloaded from Uniprot 20240216_ UP5640. For protein identification, an FDR < 0.01 was stablished in Spectronaut both at protein and peptide level. Default settings (BGS factory settings) were used for the search with additional modifications: cysteine carbamidomethylation as a fixed modification, and N-terminal acetylation and methionine oxidation as variable modifications. The precursor quantification was performed at MS2 level, and the peak area of proteotypic peptides for each protein was used for quantification.

### 2.4 Statistics

Students *t-*tests were performed to evaluate differential abundances of the proteins between distal and proximal nerve ends. All reported *p*-values are two-tailed, with *p* < 0.05 being considered as statistically significant. If an individual had multiple samples by type of nerve end and protein, median value was used.

## 3 Results

A total of 3,914 proteins were found in our dataset. A summary of these proteins with their related biological functions, cellular components and molecular functions are presented in the Supplementary Figures S1–S3.

### 3.1 Protein expression profile in the injured digital nerve ends

**Figure 2:**
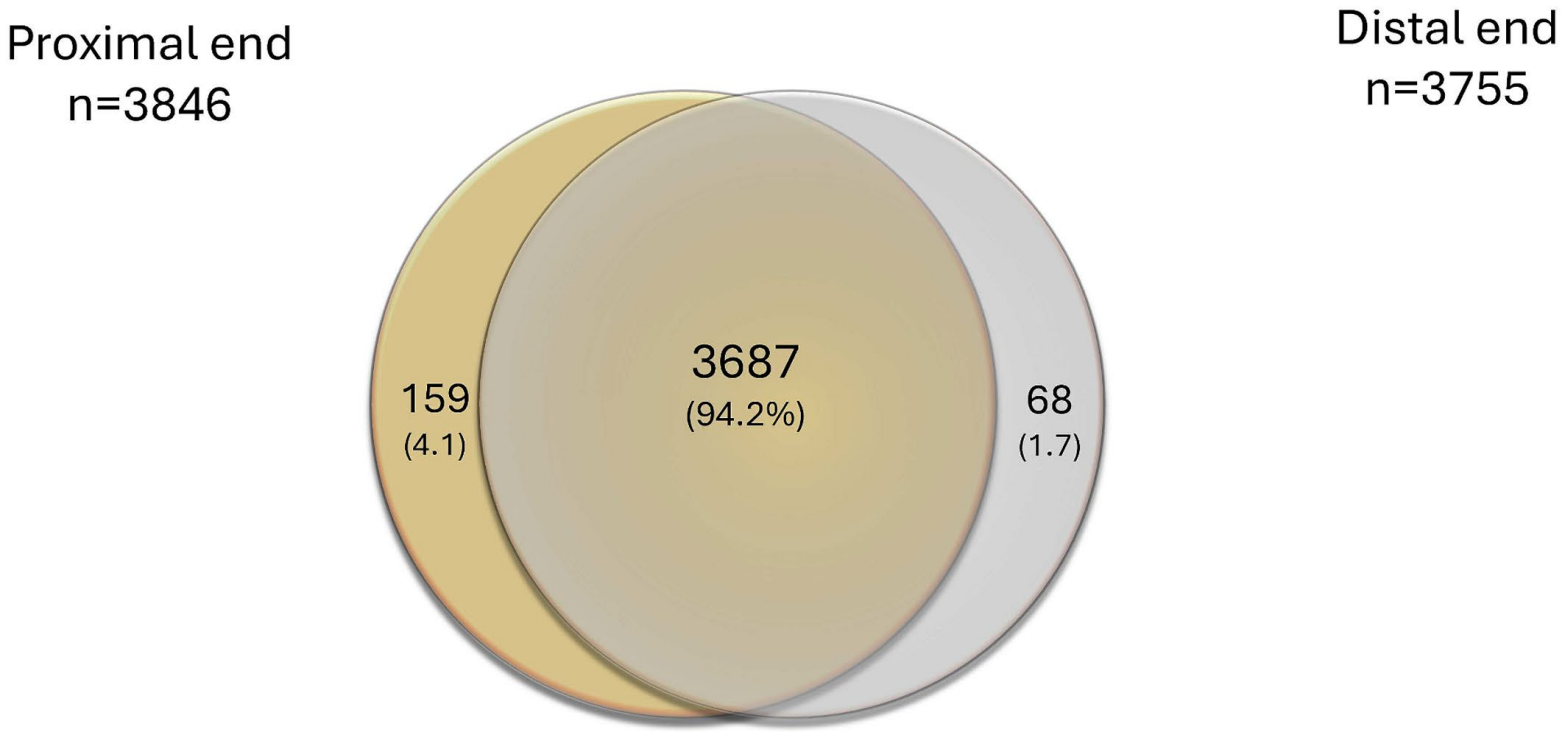
Venn diagram presenting overlaps of all identified proteins in the proximal and the distal nerve ends, alone or combined, expressed as number of proteins and % in brackets.

From the nerves of the 19 individuals analyzed, the proximal nerve end contained 3,846 proteins, while the distal nerve end consistently presented 3,755 proteins. Of those, 3,687 proteins were expressed in both nerve ends (Figure 2).

### 3.2 The difference in protein abundance between the proximal and distal nerve ends

**Figure 3:**
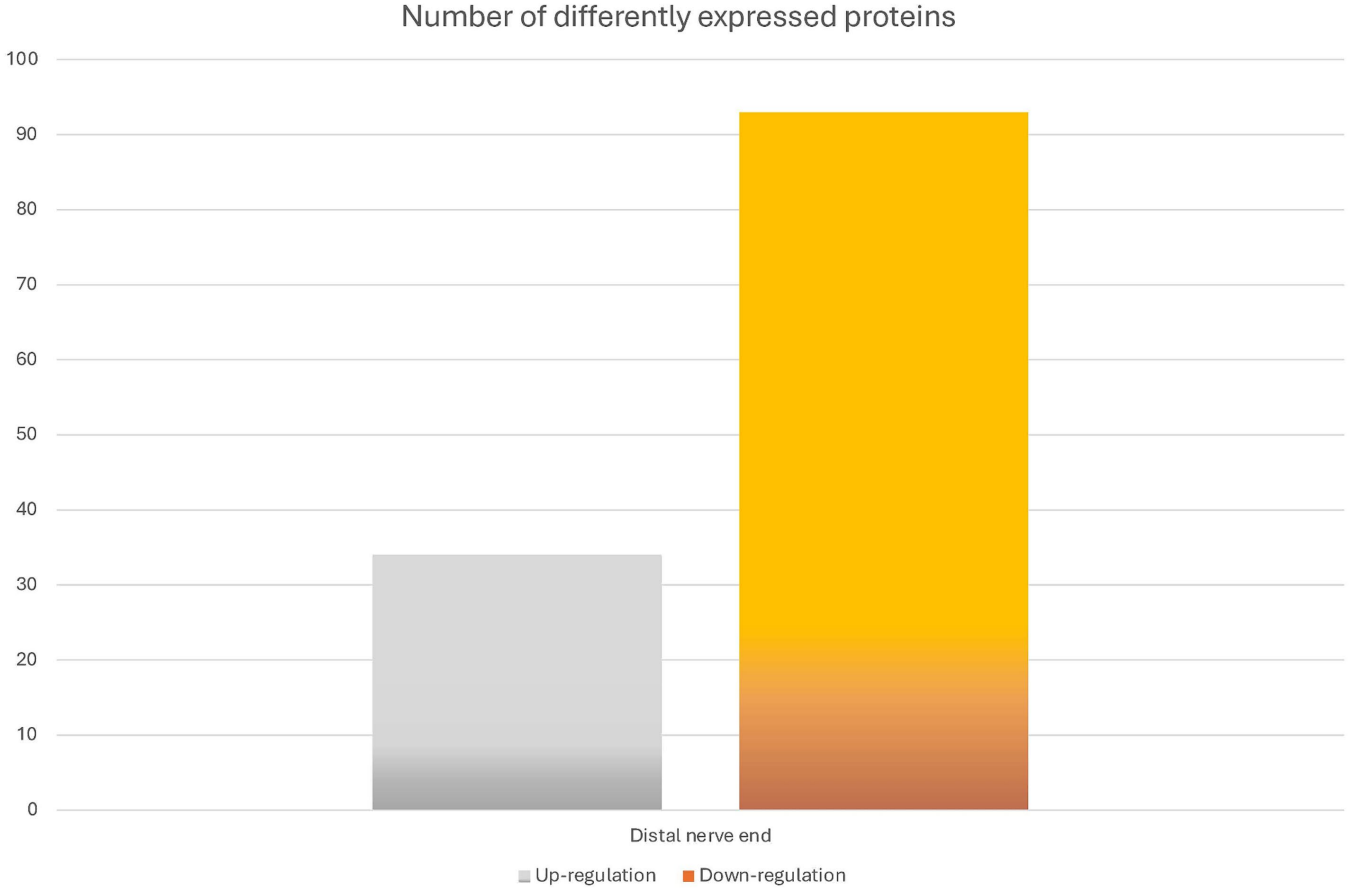
Overview of the differentially expressed proteins between the proximal and distal nerve ends. The bar graph shows the number of identified proteins that were differentially expressed when comparing the samples of the proximal (n = 26 nerve samples) and the distal nerve ends (*n* = 26 nerve samples). The difference is presented as numbers of proteins significantly either up- or down-regulated in the distal nerve end compared to the proximal nerve end with a *p*-value of less than 0.05.

To identify differentially expressed proteins in the proximal and the distal nerve ends, the relative abundance was compared between the two groups using Student’s t-test. For each group any significant changes with *p*-value of less than 0.05 were presented as up or downregulated in the distal nerve end compared to the proximal end. There were 127 proteins statistically significant up- or downregulated in the data set (Figure 3).

#### 3.2.1 Visualizing differential protein expression: volcano plot analysis of distal vs. proximal nerve ends

**Figure 4:**
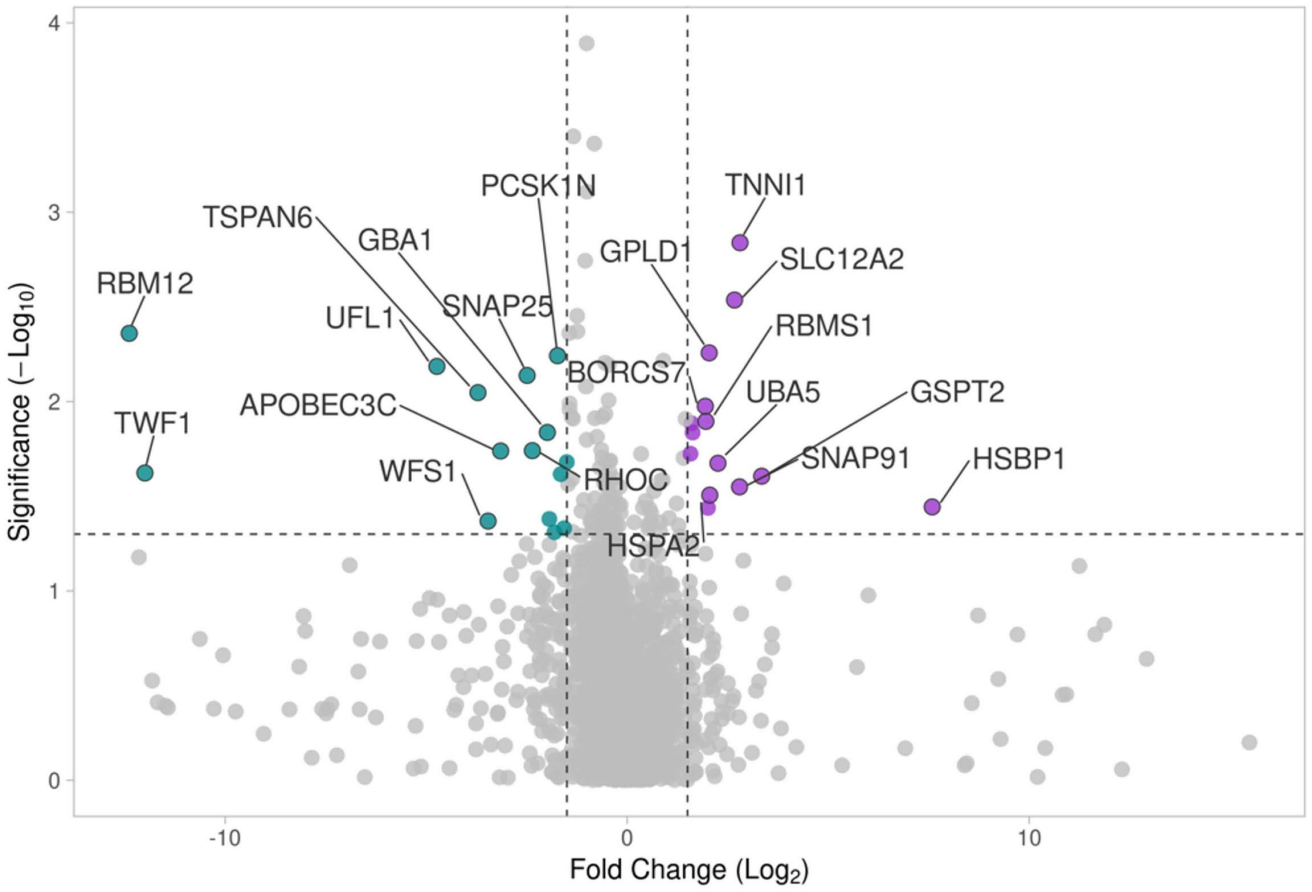
The volcano plot comparing differences in protein expression in the proximal and distal nerve ends shows log_2_ ratio versus -log_10_ *p* value of differentially expressed proteins. Colors show Up-regulated (purple) and down-regulated (blue) proteins.

A Volcano plot analysis, including a total of presently expressed 3,914 proteins between the proximal and distal nerve ends, was performed. Fold changes (x axis) were plotted against the significance -log10 *p* values (y axis) for each protein highlighting proteins with the largest and most significant differences presented in Figure 4 and in Tables 1, 2. The proteins found with the most significant differences in expression in the distal end compared to the proximal end were cross-referenced with The Human Protein Atlas (HPA) (Karlsson et al., 2021) as well as The Injured Sciatic Nerve Atlas (iSNAT) (Zhao et al., 2022) where C57BL/6 mice were studied.

**Table 1:** Proteins down-regulated in the distal nerve end, after an injury in 26 human injured digital nerves repaired within 48 h post-injury, compared with the proximal nerve end with the most substantial differences in fold change and highest significance (−log_10_ p value).

Name	Change	Fold change (log2)	Significance	Known functions	Potential role in distal nerve end	Expressed in SCs
RBM12	Decreased	−12,38	2,36	RNA binding molecule	Facilitates RNA processing and gene regulation	Yes (Karlsson et al., 2021; Zhao et al., 2022)
TWF1	Decreased	−11,99	1,623	Actin filament regulator	Controls neuronal cytoskeletal dynamics	Yes (Karlsson et al., 2021; Zhao et al., 2022)
UFL1	Decreased	−4,73	2,19	Protein quality controller	Promotes cellular integrity maintenance	Yes (Karlsson et al., 2021; Zhao et al., 2022)
TSPAN6	Decreased	−3,71	2,05	Cell membrane organizer	Guides axon membrane organization	Yes (Zhao et al., 2022)
APOBEC3C	Decreased	−3,15	1,74	RNA editing enzyme	Mediates RNA editing and immune response	Yes (Karlsson et al., 2021)
WFS1	Decreased	−3,46	1,37	ER calcium regulator	Regulates ER stress respons and calcium regulation	Yes (Karlsson et al., 2021; Zhao et al., 2022)
SNAP25	Decreased	−2,49	2,14	Vesicle fusion mediator	Facilitates neurotransmitter release	Yes (Zhao et al., 2022)
RHOC	Decreased	−2,36	1,74	Actin regulator	Regulates cytoskeletal organization	Yes (Karlsson et al., 2021; Zhao et al., 2022)
PCSK1N	Decreased	−1,73	2,24	Neuropeptide regulator	Modulates nerve function and regulation	Yes (Zhao et al., 2022)
GBA1	Decreased	−1,99	1,85	Lysosomal enzyme	Facilitates lysosomal function and neurotransmission	Yes (Karlsson et al., 2021)
HMGB3	Decreased	−1,94	1,38	Gene expression regulator	Modulates gene regulaion and inflammation	Yes (Karlsson et al., 2021; Zhao et al., 2022)
PALMD	Decreased	−1,65	1,62	Limited research	May controbute to neural function and maintenance	Yes (Karlsson et al., 2021; Zhao et al., 2022)
DBH	Decreased	−1,50	1,68	Neurotransmitter synthesis	Involved in stress response	No
MAP1LC3A	Decreased	−1,80	1,31	Autophagy	Participates in neuronal maintenance and stabilty	Yes (Karlsson et al., 2021; Zhao et al., 2022)

ER, Endoplasmic reticulum.

**Table 2:** The top 10 functional annotations (GO) Pathways for significantly up- and down-regulated proteins in the distal compared to the proximal nerve end after an injury in 26 human injured digital nerves repaired within 48 h post-injury.

Increased abundance in distal nerve end	Count	*p* value	Decreased abundance in distal nerve end	Count	*p* value
Biological process					
GO:0044699 single-organism process	30	0.015	GO:0051179 localization	48	0.002
GO:0032502 developmental process	18	0.020	GO:0044763 single-organism cellular process	73	0.003
GO:0048856 anatomical structure development	17	0.020	GO:0009987 cellular process	94	0.003
GO:0006810 transport	16	0.004	GO:0048518 positive regulation of biological process	46	0.006
GO:0051234 establishment of localization	16	0.007	GO:0044699 single-organism process	83	0.009
GO:0044765 single-organism transport	14	5.41E-4	GO:0048522 positive regulation of cellular process	40	0.022
GO:1902578 single-organism localization	14	9.81E-4	GO:0048519 negative regulation of biological process	40	0.028
GO:0006928 movement of cell or subcellular component	11	0.003	GO:0050896 response to stimulus	57	0.037
GO:1901564 organonitrogen compound metabolic process	11	0.015	GO:0071840 cellular component organization or biogenesis	57	2.22E-5
GO:0051649 establishment of localization in cell	10	0.010	GO:0016043 cellular component organization	57	8.15E-6
Cellular component					
GO:0097458 neuron part	11	4.49E-4	GO:0031982 vesicle	50	1.05E-11
GO:0044444 cytoplasmic part	28	3.01E-4	GO:0044422 organelle part	75	1.12E-6
GO:0044421 extracellular region part	12	0.042	GO:0005737 cytoplasm	79	1.58E-6
GO:0005576 extracellular region	14	0.030	GO:0044446 intracellular organelle part	75	2.32E-7
GO:0031982 vesicle	13	0.028	GO:0012505 endomembrane system	50	2.53E-9
GO:0005829 cytosol	16	0.021	GO:0005829 cytosol	45	3.80E-5
GO:0005737 cytoplasm	29	0.003	GO:0016020 membrane	67	5.46E-5
GO:0043230 extracellular organelle	11	0.003	GO:0044444 cytoplasmic part	74	6.60E-8
GO:1903561 extracellular vesicle	11	0.003	GO:0043226 organelle	87	9.10E-6
GO:0070062 extracellular exosome	11	0.002	GO:0043227 membrane-bounded organelle	83	9.70E-6
Molecular function					
GO:0005515 protein binding	31	0.009	GO:0005488 binding	93	0.001
GO:0036094 small molecule binding	11	0.010	GO:0003723 RNA binding	21	0.002
GO:000016 nucleotide binding	10	0.010	GO:0019899 enzyme binding	20	0.010
GO:1901265 nucleoside phosphate binding	10	0.010	GO:0036094 small molecule binding	21	0.030
GO:0035639 purine ribonucleoside triphosphate binding	9	0.012	GO:0097367 carbohydrate derivative binding	19	0.036
GO:0032555 purine ribonucleotide binding	9	0.014	GO:0005102 receptor binding	15	0.053
GO:0017076 purine nucleotide binding	9	0.014	GO:0044877 macromolecular complex binding	19	1.08E-4
GO:0032553 ribonucleotide binding	9	0.014	GO:0005515 protein binding	92	2.97E-9
GO:0097367 carbohydrate derivative binding	13	2.64E-4	GO:0008092 cytoskeletal protein binding	15	5.98E-4
GO:0043168 anion binding	14	6.75E-4	GO:0043168 anion binding	28	9.24E-4

##### 3.2.1.1 Proteins up-regulated in the distal nerve end

Proteins up-regulated in the distal nerve end included Heat Shock Factor Binding Protein 1 (HSBP1), Troponin I (TNNI1), Solute Carrier Family 12 Member 2 (SLC12A2), G1 to S phase Transition 2 (GSPT2), Clathrin coat assembly protein AP180 (SNAP91), Glycosylphosphatidylinositol Specific Phospholipase D1 (GPLD1), Ubiquitin Like Modifier Activating Enzyme 5 (UBA5), BLOC-1 Related Complex Subunit 7 (BORCS7), RNA Binding Motif Single Stranded Interacting Protein 1 (RBMS1), Heat Shock Protein Family A (Hsp70) member 2 (HSPA2), Integrin alpha-2 (ITGA2), Ependymin Related 1 (EPDR1), Protein FAM3C (FAM3C), and Epidermal growth factor-like protein 8 (EGFL8). All proteins except SNAP91 were found, when cross-referenced with the HPA and the iSNAT, to be expressed by SCs (Table 3).

**Table 3:** Up-regulated proteins in the distal end compared with the proximal nerve end, after an injury in 26 injured digital nerves repaired within 48 h post-injury, with the most substantial differences in expression levels and highest significance (−log_10_ p value).

Name	Change	Fold change (log2)	Significance	Known functions	Potential role in distal nerve end	Expressed in SCs
HSBP1	Increased	7,59	1,44	Heat shock response regulator	Regulation of cellular stress response	Yes (Karlsson et al., 2021; Zhao et al., 2022)
TNNI1	Increased	2,81	2,84	Muscle contraction regulator	NMJ function and mucle fibre type composition	Yes (Zhao et al., 2022)
SLC12A2	Increased	2,67	2,54	Ion transporter	Regulation of ion homeostasis	Yes (Karlsson et al., 2021; Zhao et al., 2022)
GSPT2	Increased	3,35	1,61	Protein synthesis regulator	Neuronal protein production and function	Yes (Karlsson et al., 2021; Zhao et al., 2022)
SNAP91	Increased	2,79	1,55	Endocytosis mediator	Facilitates synaptic vesicle recycling and neurotransmitter release	No
GPLD1	Increased	2,04	2,26	Lipid metabolism regulator	Promotes membrane remodeling and signaling	Yes (Zhao et al., 2022)
UBA5	Increased	2,26	1,67	Ubiquitin-like protein conjugation	Controls protein turnover and quality	Yes (Karlsson et al., 2021; Zhao et al., 2022)
BORCS7	Increased	1,94	1,98	Intracellular protein trafficker	Facilitetes organelle biogenesis and trafficking	Yes (Karlsson et al., 2021; Zhao et al., 2022)
RBMS1	Increased	1,96	1,90	RNA binding protein	Regulates RNA processing and gene expression	Yes (Karlsson et al., 2021; Zhao et al., 2022)
HSPA2	Increased	2,05	1,51	Chaperone protein	Facilitates protein folding and stress response	Yes (Karlsson et al., 2021; Zhao et al., 2022)
ITGA2	Increased	1,60	1,89	Cell adhesion molecule	Guides axon growth and synaptic connectivity	Yes (Karlsson et al., 2021; Zhao et al., 2022)
EPDR1	Increased	1,63	1,84	Neural plasticity regulator	Supports neural regeneration and plasticity	Yes (Karlsson et al., 2021; Zhao et al., 2022)
FAM3C	Increased	2,01	1,44	Intracellular signaling protein	Modulates neuronal signaling pathways	Yes (Karlsson et al., 2021; Zhao et al., 2022)
EGFL8	Increased	1,57	1,72	ECM protein	Promotes cell adhesion, migration and axonal growth	Yes (Karlsson et al., 2021; Zhao et al., 2022)

NMJ, neuromuscular junction; ECM, Extracellular matrix.

##### 3.2.1.2 Proteins down-regulated in the distal nerve end

Among the proteins down-regulated in the distal nerve end of the injured nerve compared to the proximal nerve end were Synaptosome Associated Protein 25 (SNAP25), Microtubule Associated Protein 1 Light Chain 3 Alpha (MAP1LC3A), Wolfram Syndrome 1 (WFS1), Dopamine Beta-Hydroxylase (DBH), Tetraspanin-6 (TSPAN6), RNA Binding Protein 12 (RBM12), Twinfilin Actin Binding Protein 1 (TWF1), E3 UFM1-protein ligase 1 (UFL1), DNA dC- > dU-editing enzyme APOBEC-3C (APOBEC3C), Rho-related GTP-binding protein RhoC (RHOC), Proprotein Convertase Subtilisin/Kexin Type 1 Inhibitor (PCSK1N), Glucosidase Beta Acid 1 (GBA1), High Mobility Group Protein 3 (HMGB3), and Palmdelphin (PALMD). All proteins except DBH were found, when cross-referenced with the HPA and the iSNAT, to be expressed by SCs (Table 2).

#### 3.2.2 Functional profiling of a digital nerve injury and regeneration with DAVID pathway analysis

DAVID pathway analysis (Huang da et al., 2009; Sherman et al., 2022) was performed, using the Uniprot Accession number, to group together genes with similar cell components, biological processes, and molecular functions. Of the 127 proteins, 33 proteins showed to be significantly increased in abundance at the distal nerve end, while 93 proteins were significantly decreased in the distal nerve end compared to the proximal nerve end. Table 3 presents the top 10 functional annotations (GO) pathways for the proteins significantly increased and decreased in the distal nerve end compared to the proximal nerve end.

#### 3.2.3 Functional enrichment analysis for digital nerve injury and regeneration

##### 3.2.3.1 Proteins up-regulated in the distal nerve end

**Figure 5:**
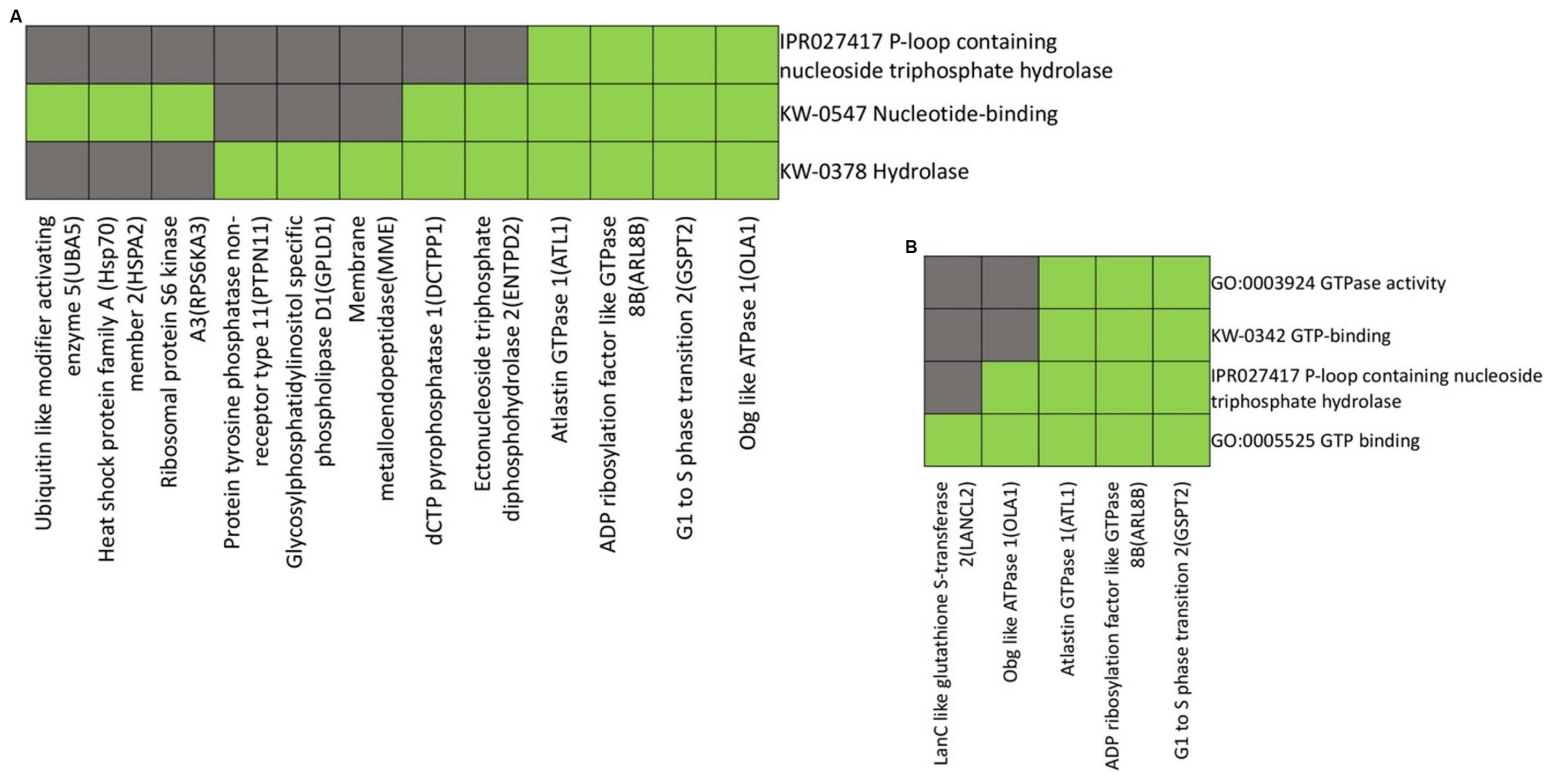
Heatmap presenting gene members and their associated annotation terms showing the gene–gene and term-term relationships within a group. Clusters **(A** and **B)** show associations of proteins up-regulated in the distal nerve end compared to the proximal nerve end with the top two highest enrichment score (< 1.0). Corresponding gene-term association positively reported presented in green, while corresponding gene-term association not reported yet is presented in black. The genes in the gene group are presented at the y axis of the heatmap, the annotation terms at the x axis.

When analyzing the functional annotations of proteins with increased abundance in the distal compared to the proximal nerve ends, we observed a clustering pattern based on shared biological functions and properties. In the cluster presented in Figure 5A, annotations reveal the common themes among Ubiquitin Like Modifier Activating Enzyme 5 (UBA5), Heat Shock Protein Family A (Hsp70) member 2 (HSPA2), Ribosomal Protein S6 Kinase A3 (RPS6KA3,) Protein tyrosine phosphatase non-receptor type 11 (PTPN11), Glycosylphosphatidylinositol Specific Phospholipase D1 (GPLD1), Membrane Metalloendopeptidase (MME), dCTP Pyrophosphatase 1(DCTPP1), Ectonucleoside Triphosphate Diphosphohydrolase 2 (ENTPD2), Atlastin GTPase 1 (ATL1), ADP Ribosylation Factor Like GTPase 8B (ARL8B), G1 to S phase Transition 2 (GSPT2), and Obg like ATPase 1 (OLA1) of nucleotide binding, hydrolase activity and P-loop containing nucleoside triphosphate hydrolase domain (Figure 5A).

The clustering proteins LanC like glutathione S-transferase 2 (LANCL2), Obg like ATPase 1 (OLA1), Atlastin GTPase 1 (ATL1), ADP Ribosylation Factor Like GTPase 8B (ARL8B) and G1 to S phase Transition 2 (GSPT2) share a common functional annotation related to GTPase activity, GTP binding as well as a P-loop containing nucleoside triphosphate hydrolase domain (Figure 5B).

##### 3.2.3.2 Proteins down-regulated in the distal nerve end

**Figure 6:**
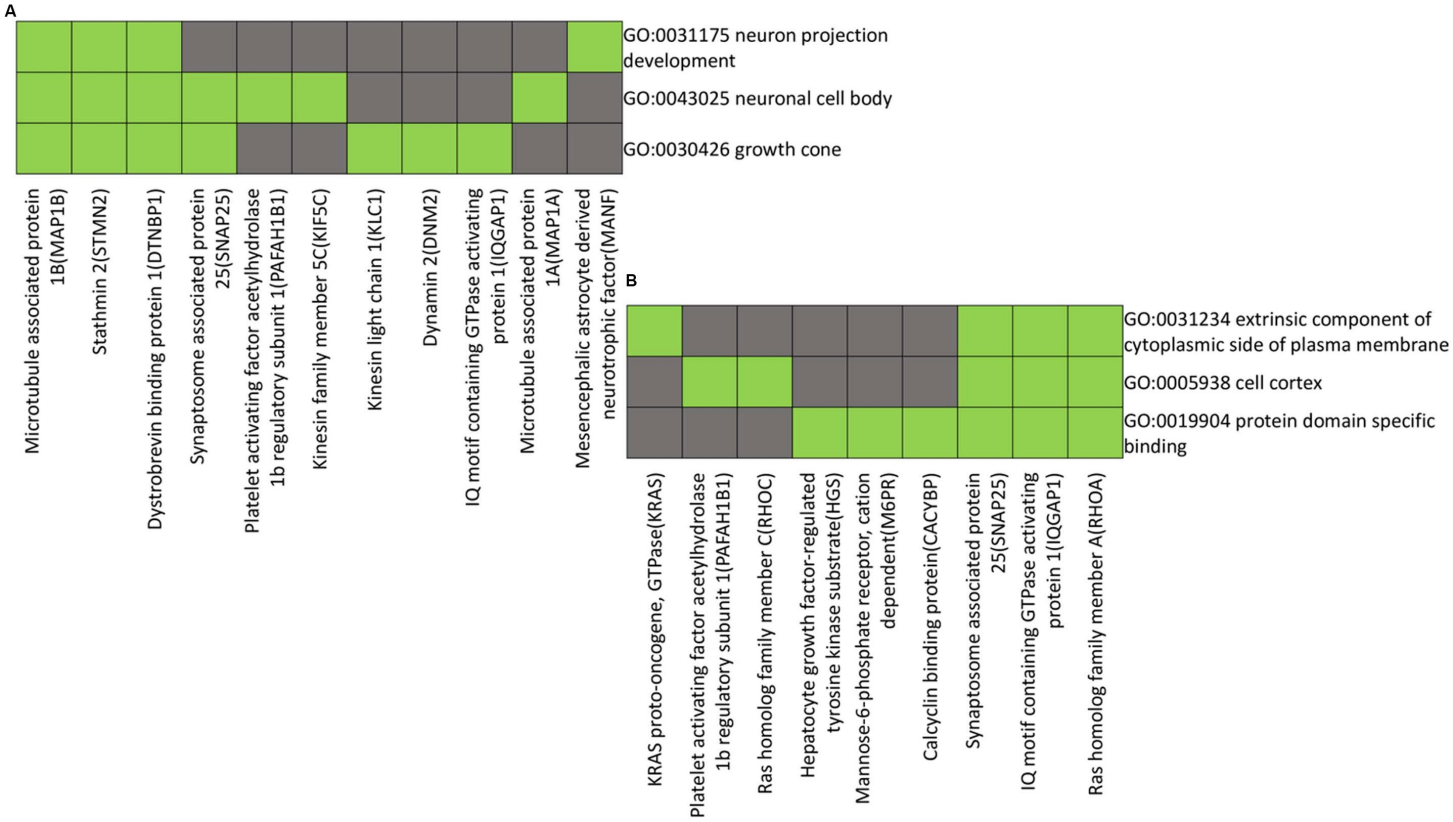
Heatmap presenting gene members and their associated annotation terms showing the gene–gene and term-term relationships within a group. Clusters **(A** and **B)** show associations of proteins with down-regulated in the distal compared to the proximal nerve ends with the top two highest enrichment score (< 2.0). Corresponding gene-term association positively reported presented in green, while corresponding gene-term association not reported yet is presented in black. The genes in the gene group are presented at the y axis of the heatmap, the annotation terms at the x axis.

The functional annotations and clustering were performed on proteins down-regulated in the distal nerve end compared to the proximal nerve end. The proteins Microtubule Associated Protein 1B (MAP1B), Stathmin 2 (STMN2), Dystrobrevin Binding Protein 1 (DTNBP1), Synaptosome Associated Protein 25 (SNAP25), Platelet Activating Factor Acetylhydrolase 1b Regulatory Subunit 1 (PAFAH1B1), Kinesin Family Member 5C (KIF5C), Kinesin Light Chain 1 (KLC1), Dynamin 2 (DNM2), IQ Motif Containing GTPase Activating Protein 1 (IQGAP1), Microtubule Associated Protein 1A (MAP1A), and Mesencephalic Astrocyte Derived Neurotrophic Factor (MANF) exhibit shared functional annotations related to neuron projection development, neuronal cell body, and growth cones, as indicated by Gene Ontology (GO) terms. (Figure 6A).

The functional annotations and clustering of proteins KRAS proto-oncogene, GTPase (KRAS), Platelet Activating Factor Acetylhydrolase 1b Regulatory Subunit 1 (PAFAH1B1), Ras Homolog Family Member C (RHOC), Hepatocyte Growth Factor-Regulated Tyrosine Kinase Substrate (HGS), Mannose-6-Phosphate Receptor, Cation Dependent (M6PR), Calcyclin Binding Protein (CACYBP), Synaptosome Associated Protein 25 (SNAP25), IQ Motif Containing GTPase Activating Protein 1 (IQGAP1), and Ras Homolog Family Member A (RHOA) show them to exhibit shared functional annotations related to the extrinsic component of the cytoplasmic side of the plasma membrane, cell cortex, and protein domain-specific binding, as indicated by Gene Ontology (GO) terms (Figure 6B).

## 4 Discussion

In this study, 3,914 proteins were identified in clear-cut injured digital nerves of the hand within 48 h post-injury. The proteomic pattern, characterized by up- and down-regulated abundances of proteins in the proximal and distal nerve ends, were investigated in connection with surgery before the nerve repair. Comparative analysis revealed 127 proteins with significant differences in regulation, with 28 proteins showing the most substantial difference between the distal and proximal nerve ends. Cross-referencing these findings with single cell RNA sequencing data from The Human Protein Atlas (HPA) (Karlsson et al., 2021) and The Injured Sciatic Nerve Atlas (iSNAT) (Zhao et al., 2022) confirmed the presences of 26 of these proteins in nerve tissue, more importantly the SCs.

During the first 48 h post-injury, the focus on the events taking place in the injured nerve is on degeneration, where axons disintegrate, and myelin sheaths break down, while SCs activate, and macrophages are recruited (Dahlin, 2023).

Our analysis of up-regulated proteins in the distal nerve end identified protein that are expressed in the axons and SCs (Karlsson et al., 2021; Zhao et al., 2022) (Table 1), as well as potentially other related cells in the nerve trunk during the process were our data and interpretation suggests significant roles in cellular processes of these cells during the nerve degeneration. The up-regulation of EGFL8, a protein highly expressed in the axons and SCs (Karlsson et al., 2021), in response to nerve injury could serve as an axon-saving mechanism facilitating early axonal outgrowth and guidance through enhanced cell adhesion, migration, and angiogenesis (Yi and Dahlin, 2010; Weiss et al., 2021) since the sprouting mechanism is initiated early. Integrin Subunit Alpha 2 (ITGA2) may guide axonal regeneration, given its role in cell adhesion and migration (Nieuwenhuis et al., 2018; Tilot et al., 2018) by promoting interactions between extracellular matrix components and guidance signals (Nieuwenhuis et al., 2018). Further, BORCS7, essential in axonal transport of lysosomes (Snouwaert et al., 2018) showed an increased expression in the distal nerve end, indicating enhanced autophagy and lysosomal degradation pathways, aiding cellular clearance processes and potentially contributing to energy conserving mechanism. Axonal transport may also continue in the distal nerve end for some time after nerve injury with accumulation of transported substances distal to the injury (Dahlin et al., 1986; Curtis et al., 1994; Lindwall and Kanje, 2005). The presence of increased SLC12A2 in the distal nerve end suggests enhanced neuronal excitability and neurotransmitter release (Ayka and Şehirli, 2020; Hu et al., 2020), crucial for synaptic plasticity and regeneration. SLC12A2, also known as NKCC1 (Zhang et al., 2021), may regulate ion transport to maintain cellular homeostasis and enhance inter-synaptic cross talk necessary for neuronal function and restoratio (Virtanen et al., 2020). Additionally, elevated HSPA2(HSP70) expression, previously identified in biopsies of the terminal branch of the posterior interosseous nerve in the forearm of type 1 diabetes (Ising et al., 2023), in the distal nerve end acting as a molecular chaperone might assist proper protein folding and degradation (Stetler et al., 2010), promoting neuronal survival and functional recovery. Its function in maintaining protein homeostasis is critical for neuronal survival and regeneration post-nerve injury (Moin et al., 2020). The UBA5 participates in activating the ubiquitin-fold modifier 1 protein (UFM1) (Fujihara and Ikawa, 2016), which regulates cellular stress responses and protein quality control mechanisms. Efficient protein quality control is important for maintaining proteostasis during nerve degeneration, and UBA5 up-regulation may enhance cellular stress responses, facilitating proper protein turnover and promoting neuronal survival.

While HSBP1 direct impact on nerve injury remains uncertain, its role in regulating the heat shock response may indicate the initial response to cellular stress immediately upon injury. It regulates heat shock factors (HSFs) promoting the expression of heat shock proteins like HSP27, crucial for protecting neurons from stress-induced injury and aiding regeneration (Almeida-Souza et al., 2011; Ylikallio et al., 2015). Given the substantial stress experienced by the distal nerve end of the injured nerve due to the nerve fiber injury and the inflammatory response, the up-regulation of HSBP1 could activate protective and survival mechanisms of the neurons and SCs facilitating cellular restoration (Collier and Benesch, 2020). Finally, FAM3C is known to be involved in metabolic regulation and resolving inflammation (Nakano et al., 2022) and may contribute to creating a supportive environment for stable nerve degeneration in the distal nerve end.

For the down-regulated proteins in the distal nerve end compared to the proximal nerve end, the proteins previously identified were found to be expressed in SCs (Zhao et al., 2022) (Table 2). This may suggest these proteins have important roles in the SCs during the nerve injury response, but their reduced abundance in the distal nerve end compared to the proximal end may indicate a functional shift or reduction. However, their alterations over time after the injury have to be clarified. Impaired axonal guidance signals in the distal nerve end may contribute to a decreased expression of TSPAN6 (Hemler, 2001) involved in cell adhesion and migration (Humbert et al., 2022). WFS1 has primarily been associated with Wolfram syndrome, a genetic disorder characterized by optic atrophy and peripheral neuropathy. While its precise involvement in nerve injury is not fully understood, its functions in calcium homeostasis and endoplasmic reticulum stress response (Takei et al., 2006) suggest potential relevance in the degeneration process because of the calcium dependent proteases needed for the degeneration process (George et al., 1995). During nerve injury calcium dysregulation can trigger various cellular processes, including activation of calpain proteases (Metwally et al., 2023), which contributes to cytoskeletal disintegration (Ma et al., 2013). Additionally cellular resources are diverted towards managing endoplasmic reticulum stress, there may be a concurrent decrease in WFS1 expression, potentially contributing to the degeneration process in the distal nerve end after injury. For SNAP25 and PCSK1N, the reduced levels of these proteins in the distal end could imply the breakdown of synaptic vesicle regulation and prohormone processing in the distal segment post-injury. SNAP25 has garnered interest not only for its role in synaptic transmission, but also for its implications in neuronal regeneration and axonal outgrowth post-injury (Osen-Sand et al., 1993; Tao-Cheng et al., 2000; Noor and Zahid, 2016; Batista et al., 2017). For RHOC, lower abundance distally might reflect specific regulatory changes in cellular adhesion and migration processes at the site of injury (Qu et al., 2019). Further HMGB3, a protein involved in DNA binding and repair, shows decreased levels distally, potentially being an indication of reduced cellular proliferation or stress responses in this region (Stros, 2010). The MAP1LC3A was among the first markers identified to associate with autophagosome structures and is the most widely used markers for autophagy (Osen-Sand et al., 1993; Yamamoto and Yue, 2014; Bonam et al., 2020). The role of autophagy in the maintenance of cell homeostasis is well documented (Bonam et al., 2020), showing that autophagy clearance mechanisms can improve the microenvironment and provide basal energy for SC survival (Huang et al., 2016). Further, alterations in autophagic activities have been linked to neuropathic pain after nerve injury (Liao et al., 2022). We lack an explanation for the observed down-regulation in MAP1LC3A, where the findings imply a potential delay in autophagic processes responsible for cellular clearance of damaged components (Osman et al., 2015; Mohseni et al., 2017), findings which have to be clarified in the future in response to nerve injury over time.

The functional annotations and clustering of up-regulated proteins in the distal nerve end suggest their association with nucleotide-binding proteins crucial roles for signaling, energy metabolism, and gene regulation. These proteins, linked to enhanced cellular signaling pathways, are involved in neuronal degeneration and restoration (The UniProt Consortium, 2017). Many of these proteins contain the P-loop nucleoside triphosphate hydrolase domain (IPR027417), indicating roles in ATPase activity (The UniProt Consortium, 2017; Kozlova et al., 2022), essential for energy metabolism, vesicular trafficking, protein folding, and cytoskeletal dynamics. Increase in ATPase activity in the injured distal nerve end supports the energy-demanding processes of nerve restoration and axonal regeneration. This up-regulation suggests an increase in energy dependent processes for nerve degeneration.

Functional annotations and clustering show that proteins down-regulated in the distal nerve end are involved in development and maintenance of sprouts and growth cones, which guide axonal growth. The down-regulation suggest that these processes have not yet begun in the distal nerve end as the nerve repair was performed after the harvest of the nerve pieces and done within 48 h post-injury. Early axonal sprouting and rapid crossing of a repaired gap can occur after nerve repair (Holmquist et al., 1993; Kerns et al., 1993; Dahlin, 2008). Proteins like MAP1B and MAP1A essential for stabilizing microtubules in growth cones and regulating axonal outgrowth, are naturally down-regulated in the distal end before axons sprouting after the nerve repair (Noiges et al., 2002; Sainath and Gallo, 2015). Similarly, STMN2, which aids in microtubule dynamics and sprout formation (Noiges et al., 2002; Krus et al., 2022) is also down-regulated. A delay in autophagy activation, important for clearing damaged cellular components, may contribute to the down-regulation of MAP1LC3A in the distal nerve end.

In the distal nerve end, proteins significantly increased linked to single-organism processes, development, anatomical structure development, and transport indicating a specialized response to nerve injury focused on tissue restoration and regeneration rather than basic cellular maintenance. This upregulation suggests effort towards rebuilding and restructuring the injured nerve tissue integral to the initial stages of nerve degeneration and subsequent restoration process.

Conversely, down-regulated proteins in the distal nerve end are associated with cellular localization, cellular processes and regulatory mechanisms. This shift likely conserves energy and resources, focusing on essential degeneration and restoration. The suppression of certain cellular activities and the reduced response to external stimuli suggest a focus on internal restoration during the degenerative phase, preparing for tissue regeneration.

Up-regulated proteins are linked to neuronal and cytoplasmic elements, indicating active neuron involvement in degeneration. Structural and functional changes in nerve cell, along with active extracellular communication, aids tissue restoration. Vesicular trafficking and secretion processes support intracellular communication (Perlson et al., 2004; Sun and Cavalli, 2010; di Paolo et al., 2021; Wu et al., 2024).

Down-regulated proteins are related to intracellular organelles and the endomembrane system, indicating decreased intracellular trafficking and organelle dynamics. Reduced membrane-bound organelles and cytosolic components reflect decreased cellular activity, conserving energy for essential restoration processes.

Molecular functions of up-regulated proteins include protein binding activities that may increase molecular interactions and signaling, important for cellular regeneration. Conversely, down-regulated proteins show reduced RNA and enzyme binding, diminishing cell signaling and cytoskeletal dynamics, and reflecting cellular quiescence during the degeneration.

The study’s specific focus on human digital nerve injuries repaired within 48 h, provides clinically relevant insights. Understanding the proteomic changes specific to human nerve injuries enhances the translational potential of the findings to future clinical practice. Moreover, the study utilized advanced mass spectrometry techniques for proteomic analysis, ensuring high sensitivity and coverage of protein identification. This integration of proteomic methodologies enhances the reliability and robustness of the study findings. We also corroborated our protein findings with the RNA sequencing databases of HPA and iSNAT to verify their expression in a peripheral nerve of mice (Karlsson et al., 2021; Zhao et al., 2022). However, the study primarily examined events during the degenerative phase of nerve injury within the initial 48 h post-injury, thereby not capturing later-stage changes or long-term outcomes associated with nerve repair and regeneration. Although the study identified proteins associated with various cellular processes and pathways, functional validation through experimental assays was not conducted. Additionally, while the study did not directly address underlying diseases, age, and potentially sex differences, these factors could influence the observed proteomic changes and warrant consideration in future investigations. Another factor to consider is the influence of the injury distance from the soma on protein expression and composition. Protein concentrations in nerve segments can vary based on their distance from the neuronal cell body due to transport dynamics of protein synthesized in the soma. Although the relatively short length of digital nerves means that variations due to distance from the soma are most likely minor, this factor may potentially introduce subtle differences in protein composition, bur the digital nerves in the fingers are located anatomically at a long distance from the related nerve cell bodies in the spinal cord and dorsal root ganglia. These limitations underscore the need for further to validate the functional roles of these proteins in nerve degeneration, regeneration, and repair or reconstruction, including the factor timing of surgery as well as investigations of proteomic gradients, which may be more relevant when addressing injuries to the major nerve trunks in the upper arm and forearm. Moreover, it is important to note that interpretation of proteomic data may be subject to bias and assumptions, potentially influencing the conclusions drawn from the study. These limitations highlight the need for further research to comprehensively understand the molecular mechanisms underlying peripheral nerve injury, degeneration, and regeneration processes, facilitating the development of more effective diagnostic and therapeutic interventions for nerve injuries.

## 5 Conclusion

Distinct differences in abundance of 127 proteins between the distal and proximal nerve ends were found within 48 h after a human digital nerve injury before nerve repair. The up-regulated proteins in the distal nerve end suggest a specialized response during the degenerative phase aimed at tissue restoration and subsequent regeneration, while down-regulated proteins indicate a shift in cellular priorities. These insights emphasize the complexity of the nerve degeneration and regeneration processes in humans, urging further detailed exploration into the underlying molecular mechanisms, particularly in relation to time after injury and timing of surgery.

## Data availability statement

The datasets presented in this study can be found in online repositories. The names of the repository/repositories and accession number(s) can be found below: http://www.proteomexchange.org/, PXD051266.

## Ethics statement

The studies involving humans were approved by Regional Ethical Review Board in Lund, Sweden (no 311/2016). The studies were conducted in accordance with the local legislation and institutional requirements. The participants provided their written informed consent to participate in this study.

## Author contributions

DF: Conceptualization, Data curation, Formal analysis, Investigation, Methodology, Project administration, Validation, Visualization, Writing – original draft, Writing – review & editing. CW: Data curation, Investigation, Methodology, Supervision, Writing – original draft, Writing – review & editing. RP: Data curation, Formal analysis, Investigation, Validation, Writing – original draft, Writing – review & editing. LD: Conceptualization, Formal analysis, Funding acquisition, Investigation, Methodology, Project administration, Resources, Supervision, Validation, Visualization, Writing – original draft, Writing – review & editing.
