# Supplementary material for: Quantitative mass spectrometry analysis of the injured proximal and distal human digital nerve ends
Source: Front Mol Neurosci. 2024 Jul 2;17:1425780. doi: 10.3389/fnmol.2024.1425780 (PMC11250671; doi:10.3389/fnmol.2024.1425780)

Supplementary Material

Quantitative Mass Spectrometry Analysis of the Injured Proximal and Distal Human Digital Nerve Ends

Drifa Frostadottir^1,2*^, Charlotte Welinder^3^, Raquel Perez ^1,4^, Lars B. Dahlin^1,2,5^.

^1^ Department of Translational Medicine – Hand Surgery, Lund University, Malmö, Sweden

^2^ Department of Hand Surgery, Skåne University Hospital, Malmö, Sweden

^3^ Faculty of Medicine, Department of Clinical Sciences, Lund, Mass Spectrometry, Lund University, Lund, Sweden.

^4^ Unit for Social Epidemiology, Department of Clinical Sciences, Malmö, Lund University, Sweden

^5^ Department of Biomedical and Clinical Sciences, Linköping University, Linköping, Sweden

*** Correspondence:**
drifa.frostadottir@med.lu.se

Keywords: Digital nerve injury, Quantitative Mass Spectrometry, Proteomics, Peripheral nerve injury, Nerve injury pathways, Signal transduction, Extracellular matrix. (Min.5-Max. 8)

**Supplemental figure 1** Top 50 Biological processes found common for the total proteins found. Orange represents the total count of proteins found and blue represent proteins found with specific biological process


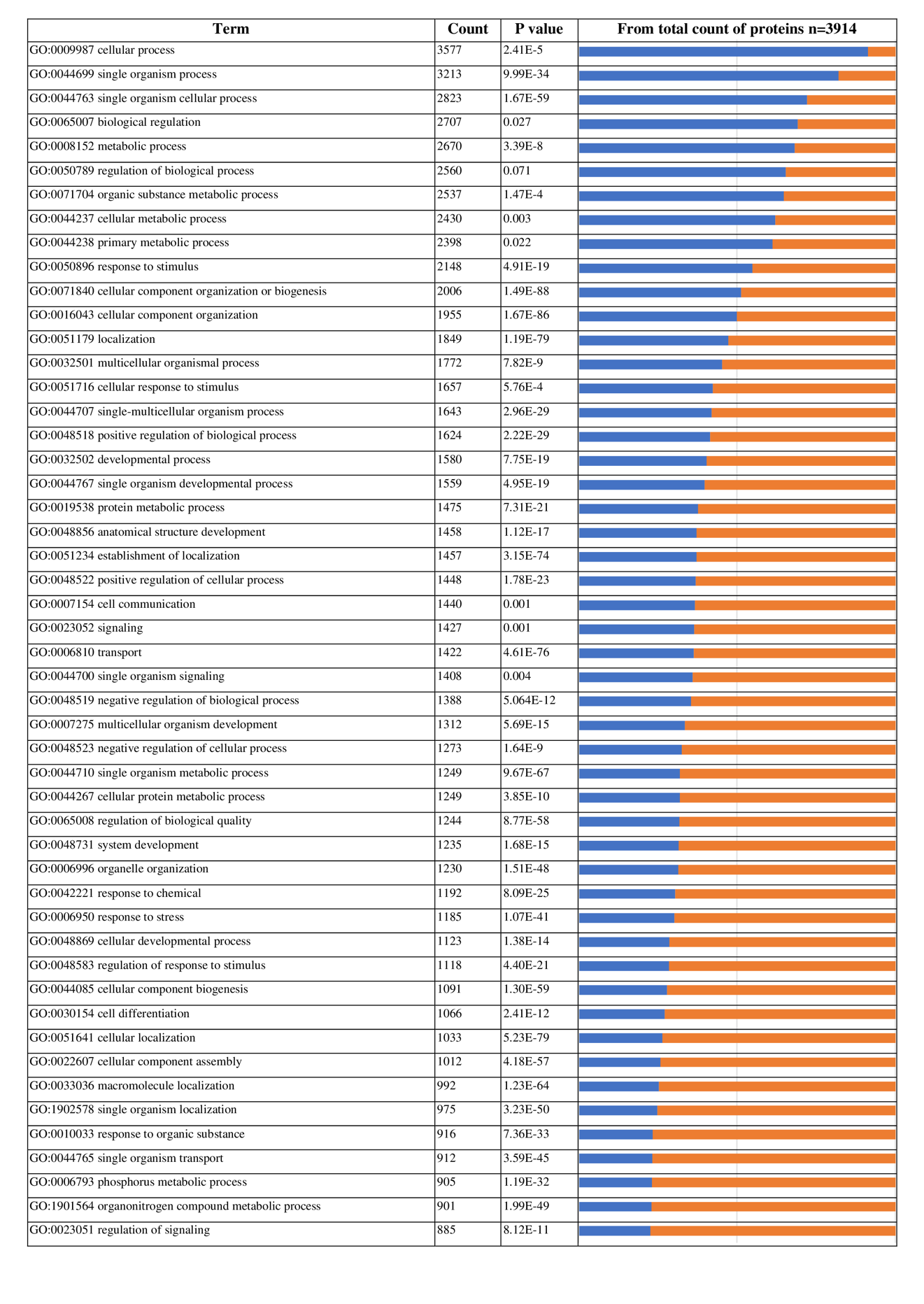


**Supplemental figure 2** Top 50 Cellular components found common for the total proteins found. Orange represents the total count of proteins and blue represent proteins found with specific cellular component


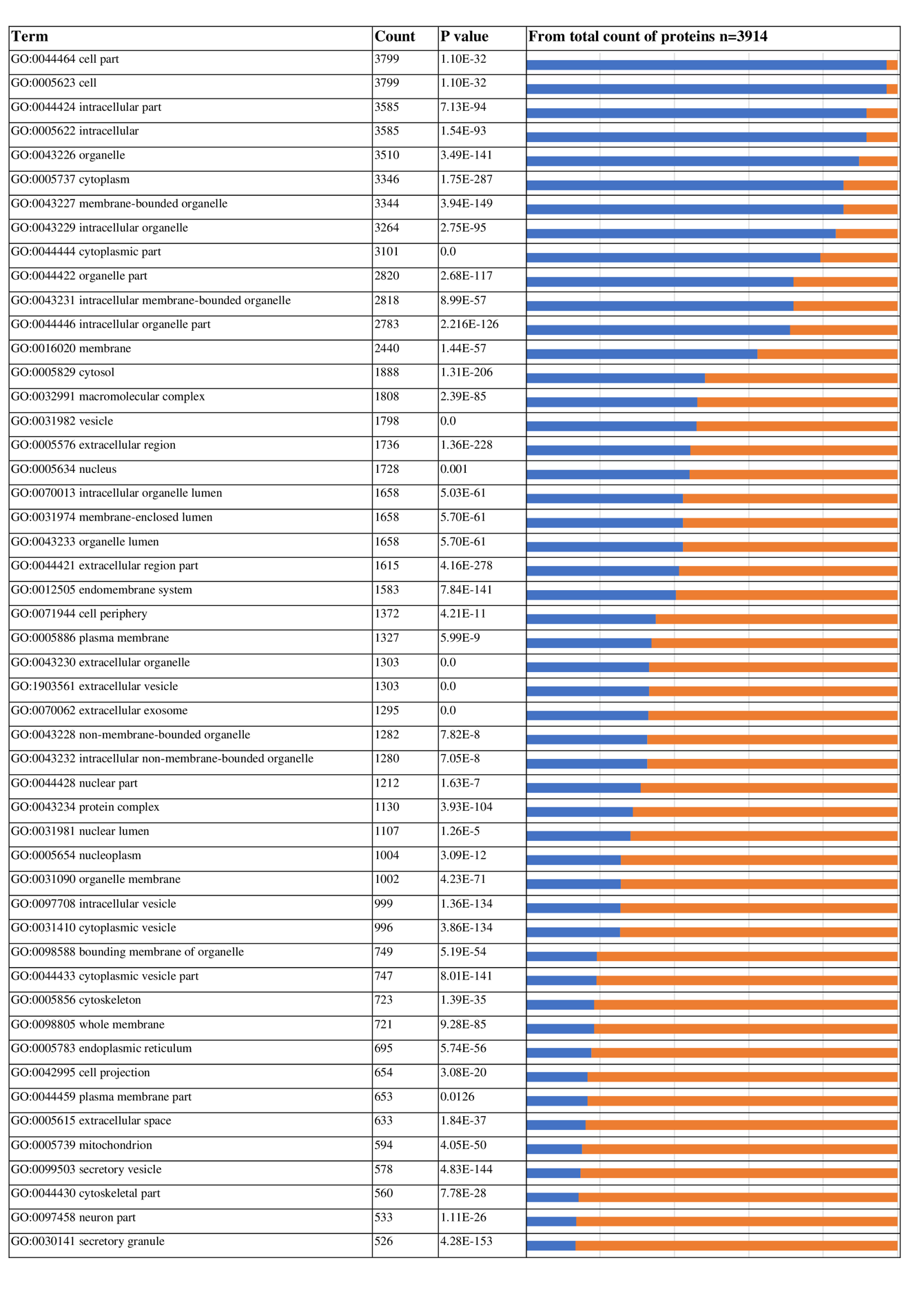


**Supplementary figure 3** Top 50 Molecular functions found common for the total proteins found. Orange represents the total count of proteins and blue represent proteins found with specific molecular function


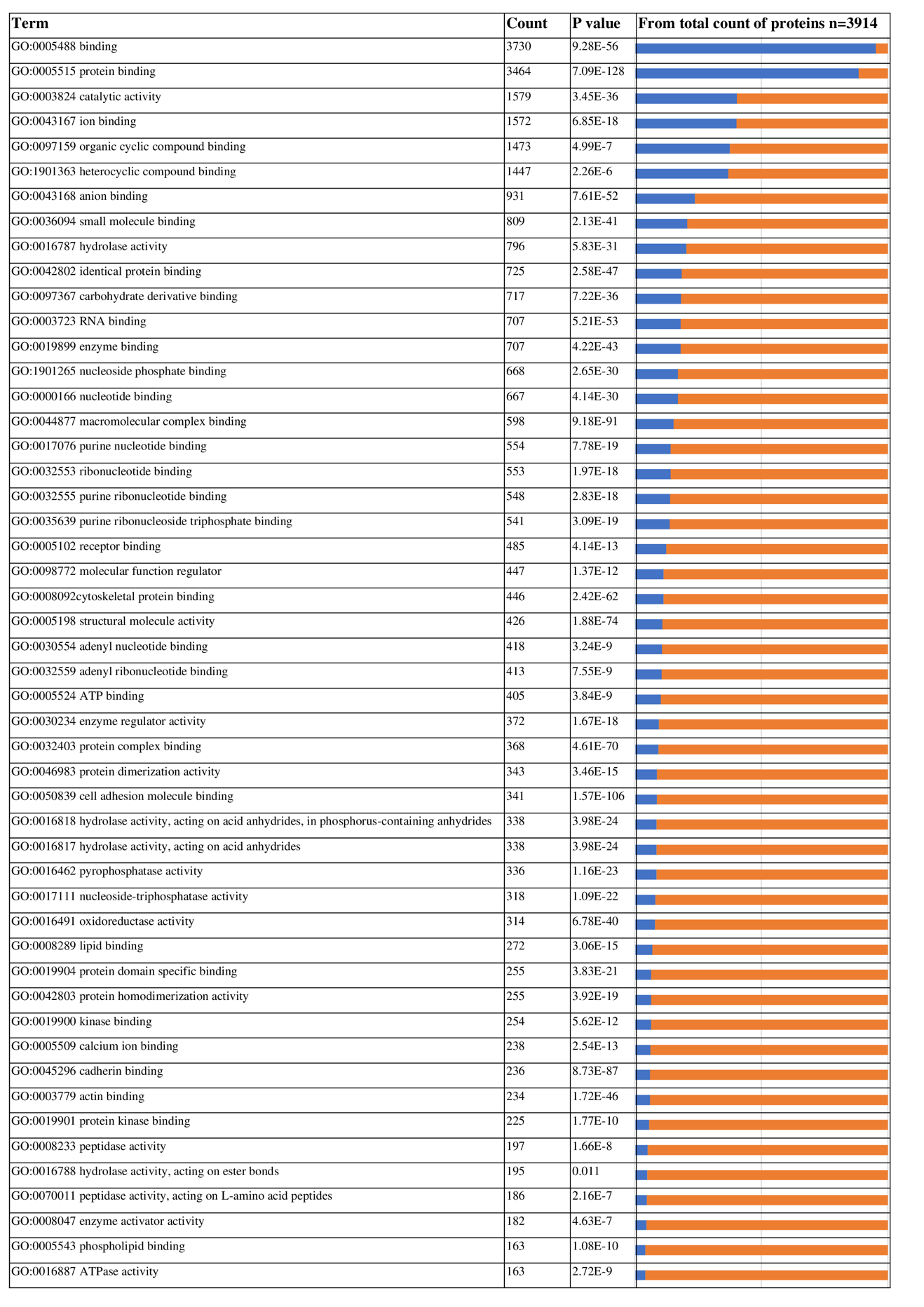

Supplement: Supplementary file 1 [file Table_1.docx]
